# Iron Oxide Nanoparticles Induce Macrophage Secretion of ATP and HMGB1 to Enhance Irradiation-Led Immunogenic Cell Death

**DOI:** 10.1021/acs.bioconjchem.4c00488

**Published:** 2024-12-16

**Authors:** Shuyue Zhan, Zhengwei Cao, Jianwen Li, Fanghui Chen, Xinning Lai, Wei Yang, Yong Teng, Zibo Li, Weizhong Zhang, Jin Xie

**Affiliations:** † Department of Chemistry, University of Georgia, Athens, Georgia 30602, United States; ‡ Department of Hematology and Medical Oncology & Winship Cancer Institute, Emory University School of Medicine, Atlanta, Georgia 30322, United States; ∥ Department of Radiology, Biomedical Research Imaging Center, and Lineberger Comprehensive Cancer Center, University of North Carolina at Chapel Hill, Chapel Hill, North Carolina 27599, United States

## Abstract

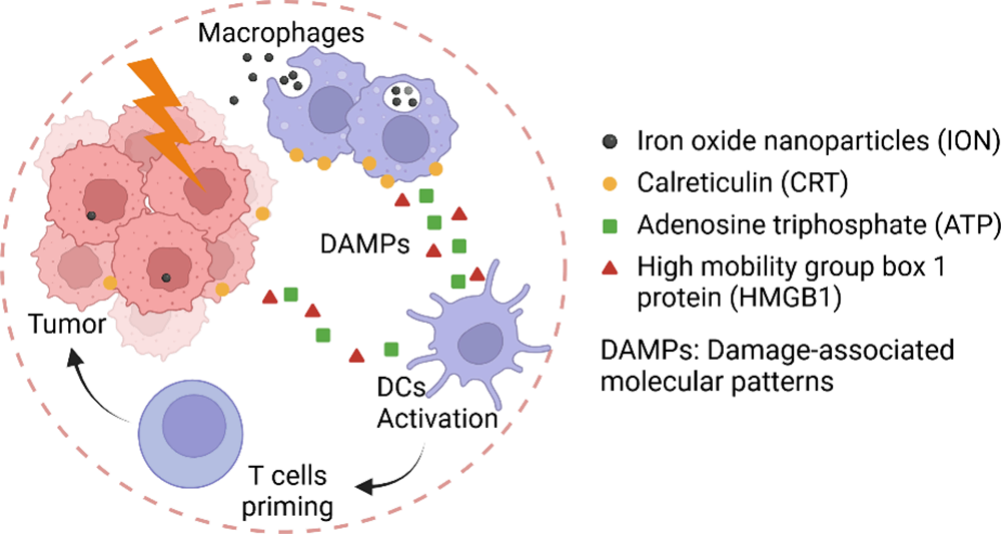

ATP (adenosine triphosphate) and HMGB1 (high mobility group box 1 protein) are key players in treatments that induce immunogenic cell death (ICD). However, conventional therapies, including radiotherapy, are often insufficient to induce ICD. In this study, we explore a strategy using nanoparticle-loaded macrophages as a source of ATP and HMGB1 to complement radiation-induced intrinsic and adaptive immune responses. To this end, we tested three inorganic particles, namely, iron oxide nanoparticles (ION), aluminum oxide nanoparticles (AON), and zinc oxide nanoparticles (ZON), *in vitro* with bone marrow-derived dendritic cells (BMDCs) and then *in vivo* in syngeneic tumor models. Our results showed that ION was the most effective of the three nanoparticles in promoting the secretion of ATP and HMGB1 from macrophages without negatively affecting macrophage survival. Secretions from ION-loaded macrophages can activate BMDCs. Intratumoral injection of ION-loaded macrophages significantly enhanced tumor infiltration and activation of dendritic cells and cytotoxic T cells. Moreover, exogenous ION macrophages can enhance the efficacy of radiotherapy. In addition, direct injection of ION can also enhance the efficacy of radiotherapy, which is attributed to ION uptake by and stimulation of endogenous macrophages. Instead of directly targeting cancer cells, our strategy targets macrophages and uses them as a secretory source of ATP and HMGB1 to enhance radiation-induced ICD. Our research introduces a new nanoparticle-based immunomodulatory approach that may have applications in radiotherapy and beyond.

## Introduction

ICD refers to the release of damage-associated molecular patterns (DAMPs) from dying cells that trigger an immune response.^1^ Cells undergoing ICD present “eat me” signals such as calreticulin (CRT) on the cell membrane while secreting “find me” signals such as ATP and “present me” signals such as HMGB1 into the tumor microenvironment (TME).^2−4^ These signals are recognized by antigen-presenting cells (APCs), primarily dendritic cells (DCs), which in turn initiate immune response.^5^ Specifically, DCs recognize ATP through the purinergic receptor P2X7 (P2RX7) and migrate toward the source.^6,23^ Binding with ATP also promotes the maturation of DCs and their release of proinflammatory cytokines.^7^ Additionally, HMGB1 acts as a strong cytokine; its binding with receptors such as toll-like receptor 4 (TLR4) induces the activation of DCs.^8^ The combined results are enhanced maturation of DCs and their migration to secondary lymphoid organs, such as lymph nodes, where they cross-present antigens to T cells. The tumor-reactive T cells then migrate to the tumor site and eliminate cancer cells through mechanisms such as cytokine release and cytotoxic effects.^9^ However, inadequate secretion of ATP and HMGB1 is often observed with cells killed by radiotherapy and chemotherapy.^10,11^ Exogenous administration of DAMPs, including ATP and CRT,^12−14^ has been shown to modulate the TME and enhance antitumor immunity. Although intriguing, these approaches have not been widely adopted, likely due to challenges such as poor targeting, rapid clearance, and unwanted toxicity.

Macrophages are a common type of immune cell that infiltrates tumors at all stages.^15^ As a major type of phagocyte, macrophages can engulf foreign microorganisms, including bacteria, viruses, and fungi.^16^ Macrophages can also ingest inorganic nanoparticles through phagocytosis, and several studies have shown that inorganic nanoparticles can affect their secretion of cytokines and chemokines.^17−22^ Recent studies suggest that exposure to inorganic nanoparticles can affect the secretion of ATP and HMGB1 from macrophages.^21,24,25^ The exact mechanisms underlying these effects are not fully understood and may vary but could be attributed to factors such as activation of inflammasomes^26^ or elevation of oxidative stress.^27^

In the current study, we explored the feasibility of using stressed macrophages as a source of ATP and HMGB1 to enhance immune responses induced by conventional therapies such as radiation. While irradiation (IR) can promote the secretion of proinflammatory cytokines such as type-1 interferon,^28,29^ radiotherapy alone is often associated with poor immunogenicity in cancer treatment. We reasoned that when exposed to metal oxide nanoparticles, macrophages can secrete DAMPs, including ATP and HMGB1, which may act as adjuvants to enhance radiation-induced immunity. We tested nanoparticles made of iron oxide,^17,30,31^ zinc oxide,^32,33^ and aluminum oxide,^34^ which have been reported by others to affect macrophage functions.^19,35,36^ Our experiments showed that all three nanoparticles can stimulate the secretion of ATP and HMGB1. Among the three, ION appears to be the most effective in terms of striking a balance between ATP/HMGB1 production and maintaining macrophage survival. In addition, we showed that secretions from iron oxide nanoparticle-treated macrophages can induce the maturation and activation of DCs. Furthermore, we demonstrate that intratumoral injection of either ION-loaded macrophages or free ION can enhance IR-induced cellular immunity.

## Results and Discussion

**Figure 1:**
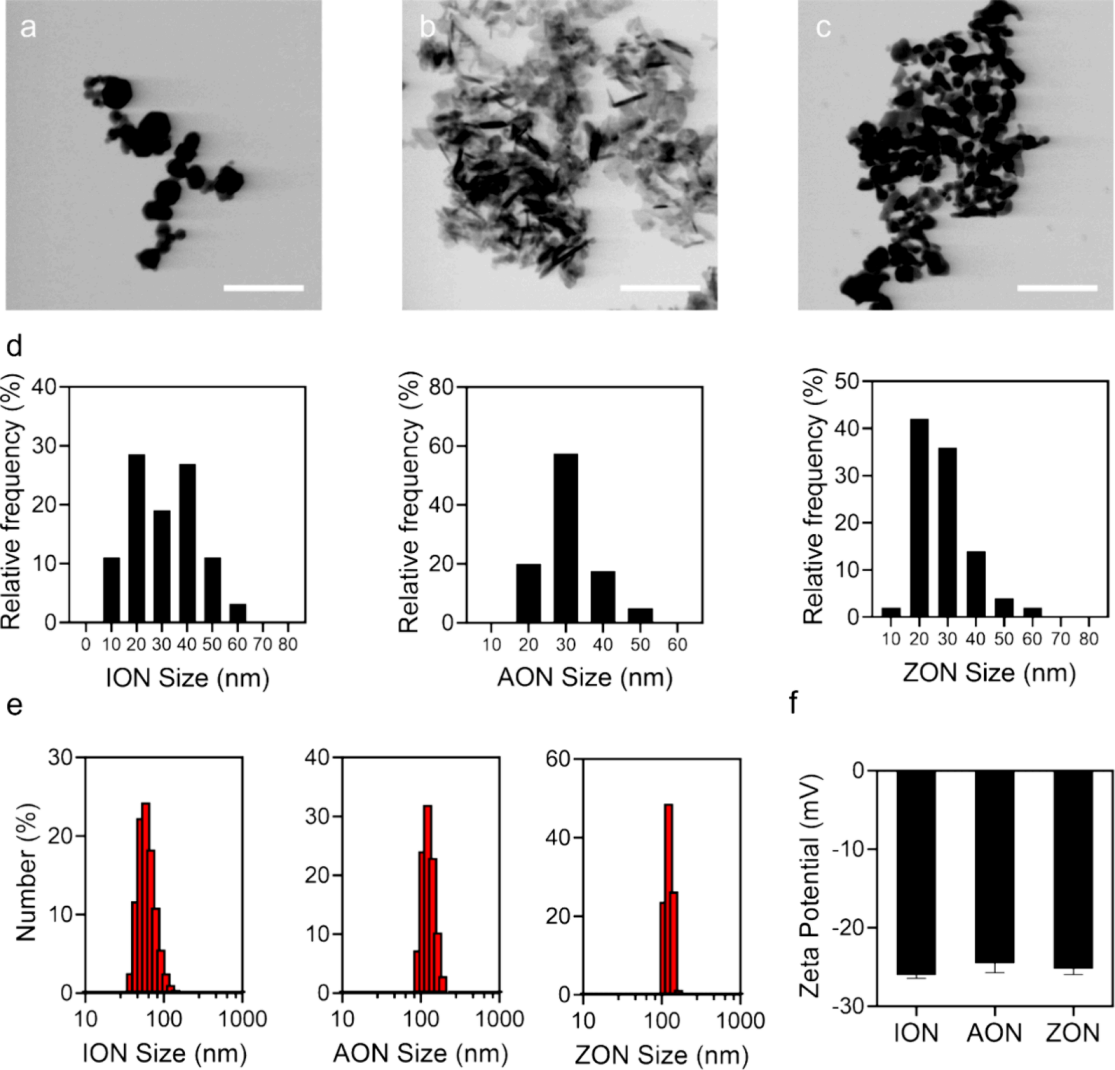
Physiochemical characterization of the three nanoparticles. TEM images of (a) ION, (b) AON, and (c) ZON. Scale bars: 200 nm. (d) Size distribution of ION, AON, and ZON, based on analysis results of TEM images using ImageJ. (e) DLS results of ION, AON, and ZON, tested in PBS. (f) Zeta potential of ION, AON, and ZON.

Fe_2_O_3_, Al_2_O_3_, and ZnO nanoparticles were purchased from commercial suppliers and used without further purification. Transmission emission tomography (TEM) revealed that all three nanoparticles are polygonal in shape (Figure 1a–c and Figure S1). The average nanoparticle sizes are 33.7 ± 10.8, 30.2 ± 7.8, and 23.1 ± 5.9 nm for ION, AON, and ZnO, respectively (Figure 1d). Dynamic light scattering (DLS) showed that the hydrodynamic sizes of the nanoparticles were 104, 200, and 236 nm (Figure 1e and Figure S2), respectively. The zeta potentials of the nanoparticle solutions in PBS (pH 7.4) are −26.1 ± 0.3 mV for ION, −24.6 ± 1.1 mV for AON, and −25.1 ± 0.8 mV for ZnO (Figure 1f).

**Figure 2:**
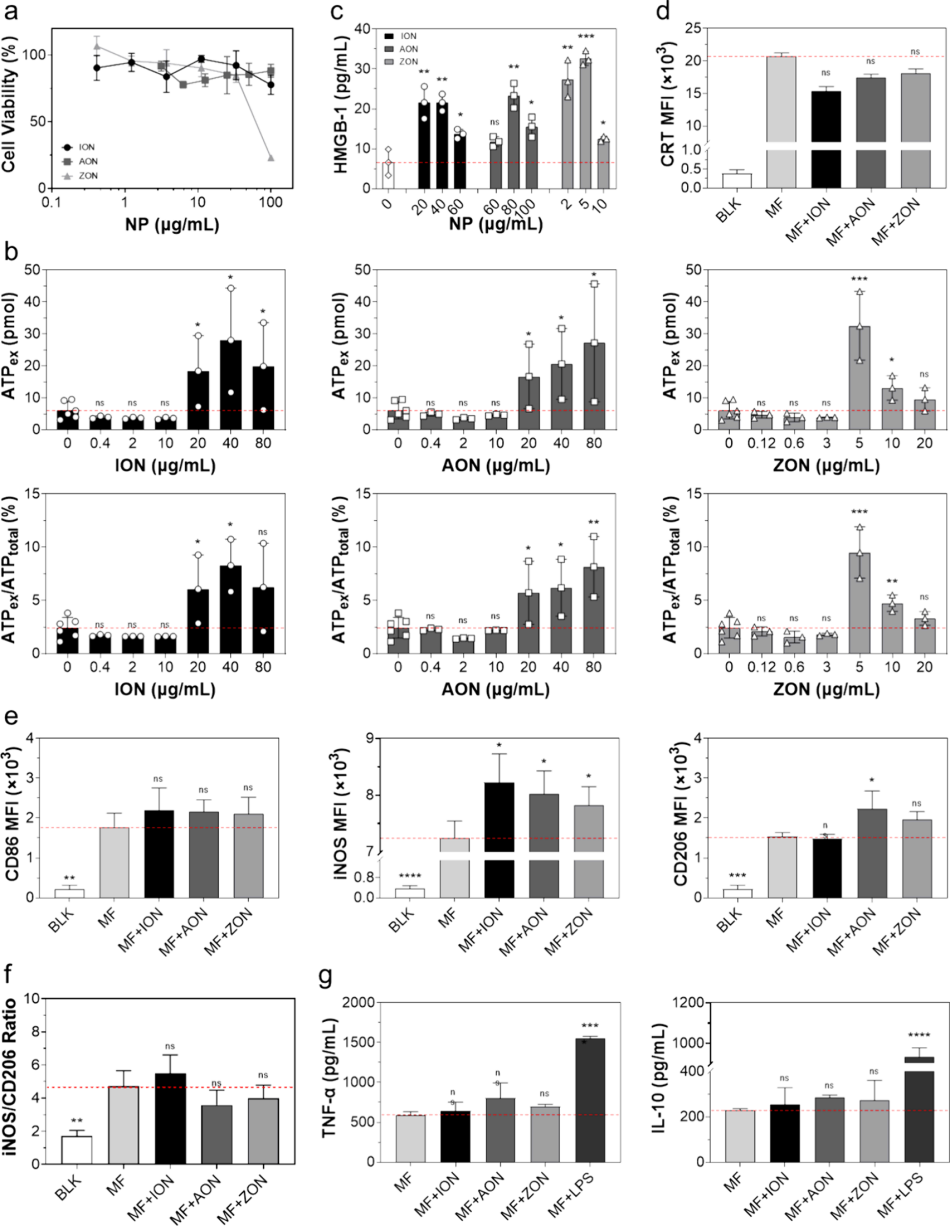
Effect of ION, AON, and ZON on macrophage survival and functions, tested *in vitro* with RAW264.7 cells. (a) Cytotoxicity, measured by the MTT assay at 24 h of incubation. (b) ATP release (upper) and the ratio between extracellular and intracellular ATP (lower), measured by a bioluminescence ATP assay. (c) HMGB1 release, measured by ELISA. (d) CRT expression, measured by flow cytometry. (e) Expression of CD86, iNOS, and CD206, measured by flow cytometry. (f) iNOS/CD206 ratio. (g) Secretion of TNF-alpha and IL-10, measured by ELISA. BLK, blank or unstained macrophages; MF, macrophages; MF+ION, MF+AON, and MF+ZON, macrophages loaded with ION, AON, and ZON, respectively. LPS treatment serves as the positive control. *p < 0.05; **p < 0.01; ***p < 0.001; ****p < 0.0001. Results are presented as means ± SD and analyzed by one-tailed *t* tests (b–d, compared to 0 μg/mL) and one-way ANOVA (e–g, compared to the MF group) with Tukey's multiple comparison test.

We examined the nanoparticles *in vitro* using RAW 264.7 cells, which are a murine macrophage cell line. All three nanoparticles showed minimal toxicity below 80 μg/mL (based on metal concentrations detected by ICP-MS). While ION and AON caused only a marginal decrease in viability even at 100 μg/mL, ZON exhibited significant toxicity at this concentration (Figure 2a). This is in accordance with observations by others finding that ZON is more toxic than ION and AON.^37,38^

We then proceeded to assess the impact of the nanoparticles on the secretion of ATP and HMGB1. All three nanoparticles promoted the secretion of ATP, but the effective concentration varies (Figure 2b). Specifically, ZON induced ATP secretion at 5 μg/mL, but the effect was almost lost at concentrations above 10 μg/mL due to toxicity. In contrast, both ION and AON significantly stimulated ATP production between 20 and 80 μg/mL (Figure 2b). Differences in HMGB1 secretion were also observed among the three nanoparticles (Figure 2c). ZON significantly induced HMGB1 secretion at 2 and 5 μg/mL, while ION and AON demonstrated similar effects at higher concentrations, between 40 and 80 μg/mL. Based on the results of both ATP and HMGB1, we chose to test ION, AON, and ZON at 40, 80, and 5 μg/mL, respectively, for further studies. It is important to note that we did not observe a significant increase in surface translocation of CRT for all three particles at these concentrations (Figure 2d), which is attributed to the sublethal doses used in our study.

Next, we examined the impact of the nanoparticles on macrophage polarization. All three nanoparticles slightly promoted the expression of CD86 and iNOS (Figure 2e),^39,40^ which are M1 markers.^41^ ION showed no effect on the expression of CD206,^42,43^ an M2 marker, whereas AON and ZON slightly promoted CD206 (Figure 2e). The iNOS^+^/CD206^+^ ratio (Figure 2f), however, remained almost unchanged during the incubation for all three nanoparticles. Consistent with the surface marker results, all three nanoparticles had little effect on the secretion of either proinflammatory cytokines such as TNF-α or anti-inflammatory cytokines such as IL-10 (Figure 2g). Taken together, these results suggest that all three nanoparticles had little effect on macrophage polarization.

**Figure 3:**
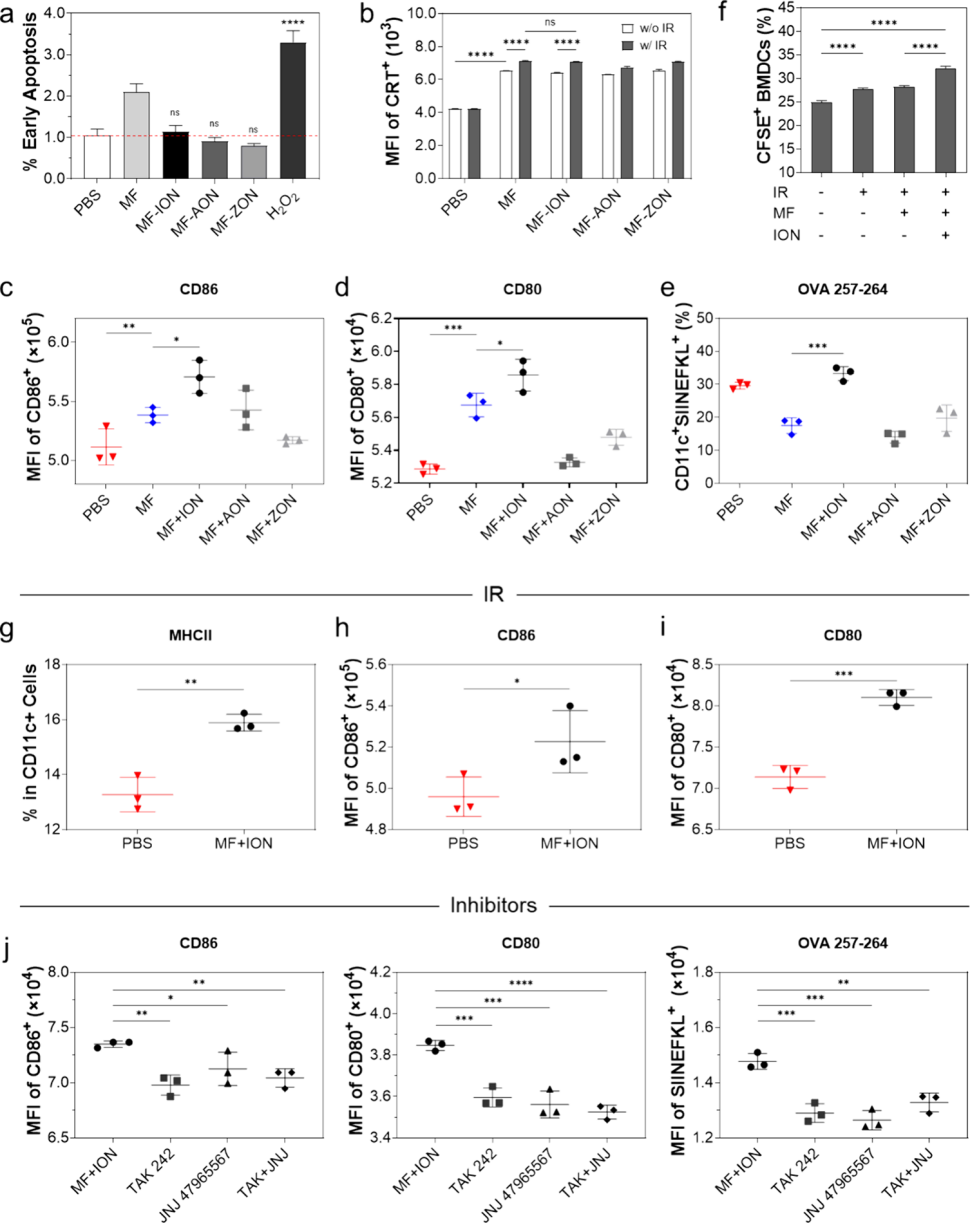
Secretions of nanoparticle-loaded macrophages affecting cancer cells and DCs. (a) Effect of secretion from macrophages pretreated with ION (MF+ION), AON (MF+AON), or ZON (MF+ZON) on the apoptosis of cancer cells (4T1), as measured by Annexin V/PI staining. (b) Effect of secretion from macrophages pretreated with ION, AON, or ZON on the expression of CRT on cancer cells (4T1), measured by flow cytometry. (c–e) Effect of secretions from nanoparticle-treated macrophages on the surface expression of (c) CD86, (d) CD80, and (e) H-2K^b^/SIINFEKL on DCs. (f) BMDC migration. Supernatants collected from nanoparticle-treated macrophages were added to the incubation medium of BMDCs. The resulting BMDCs were labeled with CFSE and loaded into the upper chamber of a transwell plate. B16F10-OVA cancer cells, exposed to 5 Gy radiation or not, were seeded in the lower chamber. (g–i) BMDCs were cocultured with irradiated (5 Gy) B16F10-OVA cells. Effect of the secretion from ION-loaded macrophages on DC maturation as assessed by flow cytometry by measuring the surface expression of (g) MHC-II, (h) CD86, and (i) CD80. (j) Effect of TAK-242, a TLR 4 inhibitor, and JNJ 47965567, a P2X7 antagonist, on DC maturation. Treatment conditions were otherwise the same as those in g–i. *p < 0.05; **p < 0.01; ***p < 0.001; ****p < 0.0001. Results are presented as means ± SD and analyzed by one-way ANOVA (a,c–f,j) with Tukey’s multiple comparison test, two-way ANOVA (b) with Šídák’s multiple comparison test, and one-tailed Student’s *t* test (g–i).

We then investigated whether macrophages under the influence of the nanoparticles affect cancer cell growth. We took supernatants from a culture of nanoparticle-treated macrophages and added them to the incubation medium of 4T1 cells. Annexin V/PI staining showed no evidence of increased cell apoptosis (Figure 3a), indicating that the conditioned media from nanoparticle-loaded macrophages had no direct effect on cell survival. We also examined the effect of macrophage secretions on cancer cell surface CRT, with or without preirradiation applied to cancer cells (5 Gy, X-ray). We found that the presence of macrophages resulted in the promotion of CRT expression on the surface of cancer cells (Figure 3b). However, we observed no difference between macrophages and nanoparticle-loaded macrophages, indicating that ION, AON, or ZON did not enhance the ability of macrophages to induce surface exposure of CRT on cancer cells.

Next, we investigated whether nanoparticle-loaded macrophages affect DC activation with BMDCs. Briefly, we first incubated RAW264.7 cells with ION, ZON, or AON and then transferred the supernatant to the incubation medium of BMDCs. We found that the conditioned medium from ION-loaded macrophages, but not from AON- or ZON-loaded macrophages, induced DC maturation, marked by an increased expression of CD86 (Figure 3c) and CD80 (Figure 3d). Next, we evaluated the effect on antigen presentation using a coculture of B16F10-OVA cells and BMDCs. The results suggest that secretion from ION-loaded macrophages, but not those loaded with AON or ZON, promotes the expression of H-2K^b^/SIINFEKL on the surface of DCs (Figure 3e). Based on these results, we have decided that ION was the most effective of the three nanoparticles to enhance DC maturation and was used in the subsequent assays.

We also investigated whether ION-loaded macrophages could affect DC migration. This was investigated using a transwell assay in which B16F10-OVA cells, irradiated (5 Gy) or not, were seeded in the lower chamber, while BMDCs were loaded into the insert and incubated with macrophage secretions (Figure 3f). IR alone promoted DC migration, which was attributed to IR-induced secretion of chemokines and “find me” signals such as ATP from cancer cells.^44,45^ While untreated macrophages had little effect, ION-treated macrophages further enhanced the migration of DCs toward irradiated cancer cells (Figure 3f). We collected cells from the lower chamber and analyzed the DC (CD11c^+^) population by flow cytometry. We found that secretion from ION-treated macrophages significantly enhanced DC maturation, as manifested by an increased population of MHC-II^+^ cells (Figure 3g) and an increased expression of surface CD86 (Figure 3h) and CD80 (Figure 3i).

ATP activates DCs by binding to the P2X7 receptor, whereas HMGB1 is recognized by DCs through TLR4. We found that TAK-242, a TLR4 inhibitor, and JNJ 47965567, a P2X7 antagonist, can both inhibit DC activation by ION macrophages, as evidenced by reduced expression of CD86 and CD80, and decreased antigen presentation (Figure 3j). These results support that ATP and HMGB1 released by ION macrophages contribute to DC activation.

**Figure 4:**
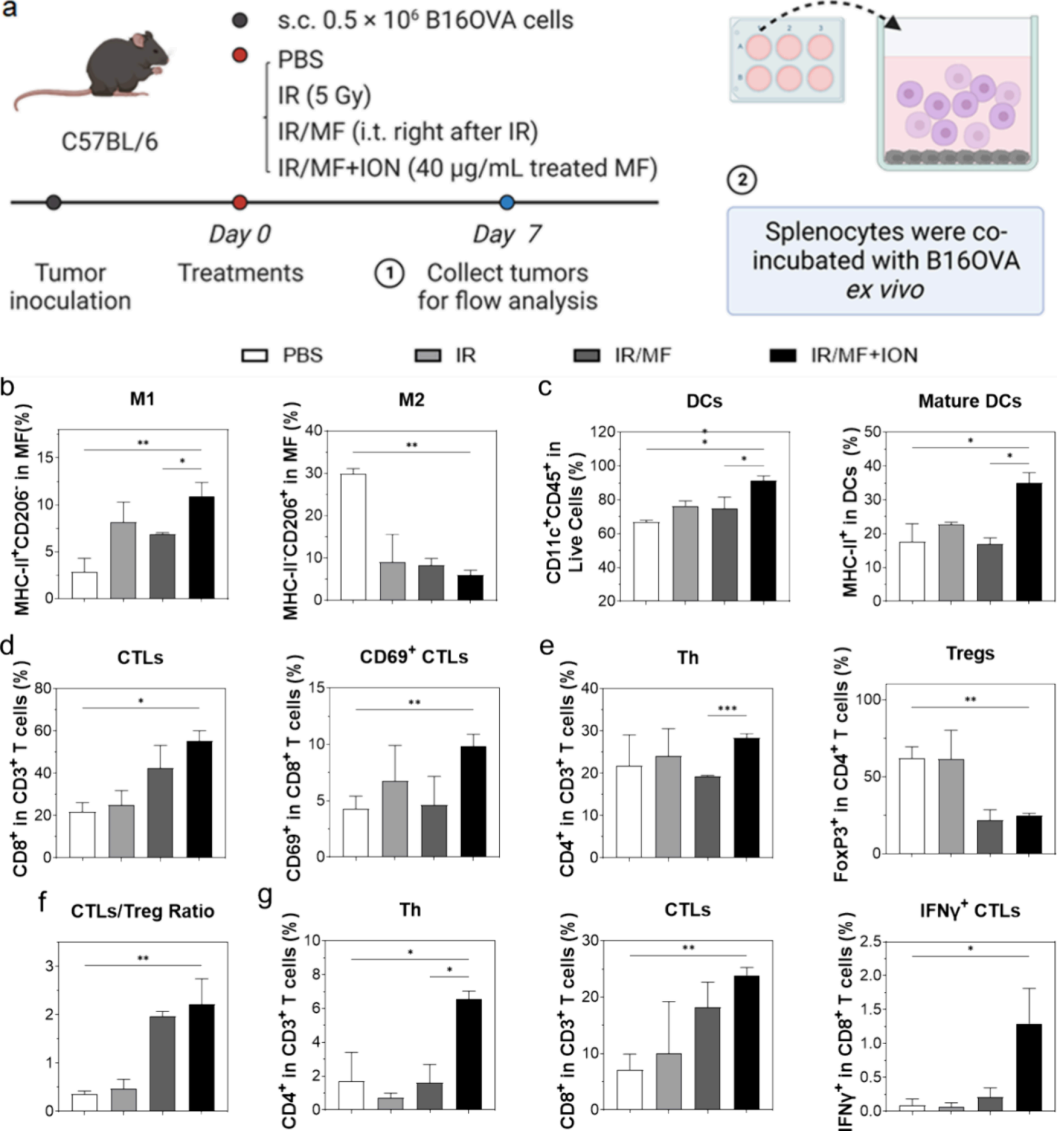
Impact of ION-loaded macrophages on the TME. (a) Schematic representation of the experimental design. B16F10-OVA tumor-bearing mice received IR (5 Gy) applied to tumors. Bone marrow-derived macrophages (MF) or ION-loaded macrophages (MF-ION) were administered i.t. immediately after the irradiation. Animals were sacrificed after 7 days. Tumors were harvested, and the immune cells were profiled by flow cytometry. In addition, splenocytes were collected and cocultured with B16F10-OVA cells *ex vivo* to evaluate the antigen-specific immune response. (b) M1 (MHC-II^+^F4/80^+^) and M2 (CD206^+^ F4/80^+^) macrophages in tumors. (c) Tumor-infiltrating DCs (CD11c^+^) and mature DCs (MHC-II^+^CD11c^+^). (d) Tumor-infiltrating CTLs (CD3^+^CD8^+^) and effector CTLs (CD3^+^CD8^+^CD69^+^). (e) T helper (Th, CD3^+^CD4^+^) cells and Tregs (CD3^+^CD4^+^FoxP3^+^) in the tumor. (f) CTL/Treg ratio. (g) *Ex vivo* stimulation of splenocytes with B16F10-OVA cells. The populations of Th, CTLs, and effector CTLs were evaluated by flow cytometry. *p < 0.05; **p < 0.01; ***p < 0.001; ****p < 0.0001. Results are expressed as means ± SEM and analyzed by one-way ANOVA with Tukey’s multiple comparison test.

We next investigated the effect of ION-loaded macrophages on the TME. To test this, we incubated ION with bone marrow-derived macrophages (BMDMs), and after washing, administered the ION-loaded BMDMs intratumorally (i.t.) to B16F10-OVA tumor-bearing C57BL/6 mice (Figure 4a and Figure S4). For comparison, untreated BMDMs were tested. Prior to macrophage injection, tumors were irradiated (5 Gy) to trigger tumor antigen release. We euthanized the animals after 1 week and harvested tumors for analysis by flow cytometry. We found that IR increased the population of M1 macrophages in tumors while reducing intratumoral M2 macrophages compared to untreated tumors (Figure 4b). Administration of macrophages did not alter the populations of M1 and M2 macrophages in the tumor or the M1/M2 ratio (Figure S5). In addition, exogenous macrophages had little effect on the infiltration of DCs, including mature (MHC-II^+^) DCs (Figure 4c). Macrophage injection increased the infiltration of cytotoxic T cells (CTLs) but did not increase activated T cells (CD69^+^ CTLs) (Figure 4d).

In comparison, injection of ION-loaded BMDMs increased the M1 population in the tumor on day 7 (Figure 4b) and increased the intratumoral M1/M2 ratio compared to untreated macrophages (although not significantly). ION-loaded macrophages also increased the amount of mature DCs (Figure 4c) and activated CTLs (Figure 4d) compared to untreated macrophages. In addition, while administration of ION-loaded macrophages had little effect on the total number of T helper cells (Figure 4e), it reduced the frequency of regulatory T cells (Tregs) in tumors (Figure 4e) and increased the CTL/Treg ratio (Figure 4f). Taken together, these results suggest that ION-loaded macrophages altered the TME to a more favorable proinflammatory state.

In addition, we harvested spleens from all treatment groups and analyzed antigen-specific immunity by restimulating splenocytes with B16F10-OVA cells and measuring activated CTLs by flow cytometry. We found an increased CTL population in splenocytes from the ION macrophage group compared to the untreated macrophage group (Figure 4g). More importantly, we saw a marked increase in the frequency of IFNγ^+^ CTLs (Figure 4g) in the ION macrophage group. All these results support that ION loading prior to macrophage injection enhances antigen-specific cellular immunity.

**Figure 5:**
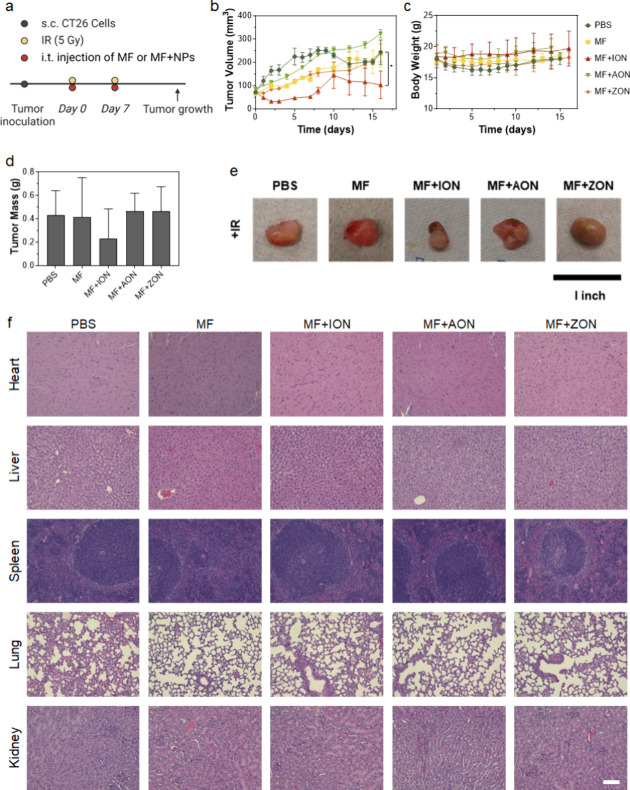
Therapeutic effects of nanoparticle-loaded macrophages, evaluated in CT26 tumor-bearing Balb/c mice. Tumors were irradiated (5 Gy) followed by i.t. administration of PBS, RAW264.7 cells (MF), or ION-, AON-, and ZON-loaded RAW264.7 cells (designated as MF-ION, MF-AON, and MF-ZON, respectively). A total of two treatments were administered 7 days apart. Animals were euthanized 16 days after the first treatment (*n* = 5). (a) Schematic representation of the experimental design. (b) Tumor growth curves. Average tumor growth. *p < 0.05; **p < 0.01; ***p < 0.001; ****p < 0.0001. Results are presented as means ± SD and analyzed by one-tailed *t* test. (c) Body weight curves. (d) Weight of dissected tumors. Results are expressed as means ± SD. (e) Representative photographs of dissected tumors from each group. (f) H&E staining of major organ tissues. Scale bar: 100 μm.

We then evaluated the therapeutic effect of ION-loaded macrophages. We hypothesize that administration of ION-loaded macrophages can improve the efficacy of radiotherapy by altering the TME and enhancing immunity. We first tested this in CT26 tumor-bearing Balb/c mice (Figure 5a). We found that animals treated with radiation (5 Gy on day 0 and day 7) plus ION-loaded macrophages (i.t.) showed reduced tumor growth compared to radiation alone (Figure 5b,d,e). Impressively, two-thirds of the animals in the radiation plus ION macrophage combination group showed tumor regression after the second macrophage administration. The treatments did not cause any weight loss (Figure 5c) or signs of acute toxicity in the animals. H&E staining showed no evidence of side effects on major organs, indicating minimal systemic toxicity (Figure 5f). For comparison, we also tested AON- and ZON-loaded macrophages. In contrast to ION-loaded macrophages, AON- and ZON-loaded macrophages showed little therapeutic benefit (Figure 5b,d,e).

**Figure 6:**
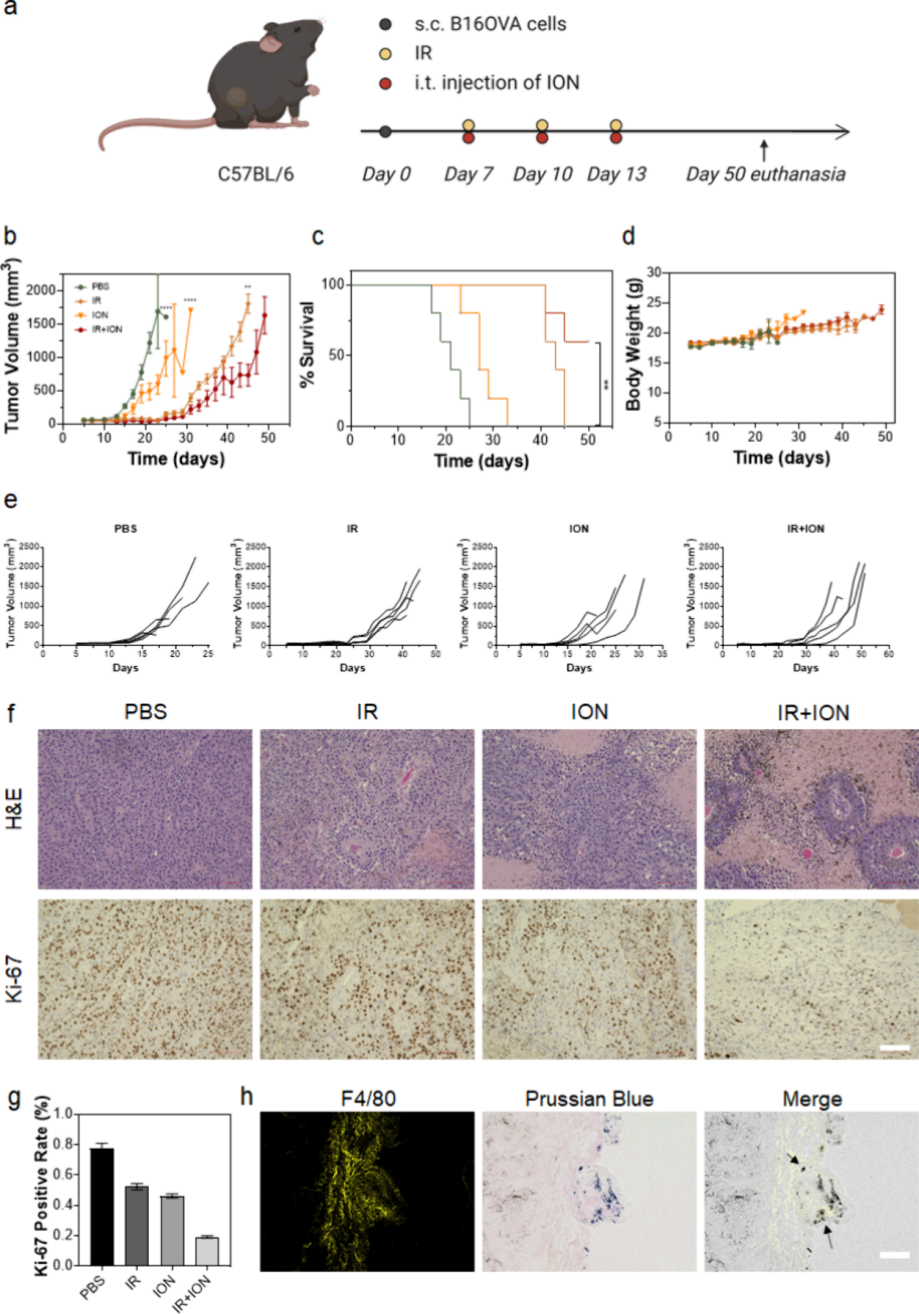
Therapeutic effects of ION in combination with IR, evaluated in B16F10 tumor-bearing C57BL/6 mice. (a–g) Evaluation of the therapeutic benefit. Seven days after inoculation, the animals received a total of three doses of treatment 2 days apart. These included PBS alone, IR (5 Gy, IR), ION alone (ION, 0.1 mg), and a combination of IR and ION (IR+ION) (*n* = 5). (a) Schematic representation of the experimental design. (b) Average tumor growth. *p < 0.05; **p < 0.01; ***p < 0.001; ****p < 0.0001. Results are presented as means ± SD and analyzed by one-tailed *t* tests. (c) Animal survival. **p < 0.01, analyzed by long-rank (Mantel–Cox) tests. (d) Animal body weight changes. (e) Individual tumor growth curves. (f) H&E (upper) and Ki-67 (lower) staining of tumor tissues. Scale bar: 100 μm. (g) Positive staining rate of Ki-67, based on results from (f). Therapeutic effects of ION in combination with IR, evaluated in B16F10 tumor-bearing C57BL/6 mice. (h) Uptake of ION by endogenous macrophages, analyzed by Prussian blue and F4/80 double staining. Scale bar, 100 μm.

A previous study has shown that i.t. administered ION is mainly taken up by macrophages in tumors.^46^ Indeed, Prussian blue and F4/80 double staining confirmed that many of the i.t. injected ION were taken up by macrophages (Figure 6h). We hypothesize that like exogenously introduced ION macrophages, endogenous macrophages may alter the TME after uptake of ION, thereby enhancing radiotherapy. We tested this again in B16F10 tumor-bearing C57BL/6 mice (Figure 6a). Briefly, we irradiated tumors (5 Gy) and administered ION i.t. after 1 h. A total of three treatments were administered 2 days apart. ION alone had a marginal to moderate effect on tumor growth compared to the vehicle alone (Figure 6b,e). Radiation was effective initially, but the treated tumors experienced relapse after 3 weeks. The addition of ION to the regimen improved the efficacy of radiation and significantly improved tumor suppression (*p* = 0.0025 on day 45). A significant increase in animal survival was also observed (Figure 6c). Histopathology data showed a reduced cancer cell population (Figure 6f) and a reduced frequency of proliferating cells (Figure 6g), also supporting the benefits of combining ION with radiation. Meanwhile, no drop of body weight (Figure 6d) nor adverse effects on major organs were observed (Figure S8).

## Conclusions

In this study, we compared the ability of three inorganic nanoparticles to stimulate the secretion of ATP and HMGB1 from macrophages. Among the three nanoparticles evaluated, ION proved to be the most effective in modifying macrophage secretions, particularly in the context of promoting activation and migration of DCs. When evaluated *in vivo*, ION-loaded macrophages outperformed AON- and ZON-loaded ones in enhancing the efficacy of radiotherapy. In addition, direct administration of ION was taken up by endogenous macrophages, resulting in enhanced tumor suppression and animal survival following radiation.

While others have investigated the interaction between inorganic nanoparticles and macrophages, the focus has often been on macrophage polarization and secretion of cytokines such as IL-1β.^18,19^ In contrast, our research revealed significant nanoparticle-induced ATP and HMGB1 secretion and its effect on DCs. Metal oxide nanoparticles after phagocytosis are expected to accumulate in the endosomes of macrophages, and the positive charged surface may facilitate the process.^47^ Previous studies have suggested that these nanoparticles may activate inflammasomes, which are involved in ATP and HMGB1 secretion.^26^ In addition, inorganic nanoparticles may increase oxidative stress,^27^ which in turn induces autophagy,^48^ leading to ATP secretion.^49^ We did not observe a significant increase in reactive oxygen species (ROS) with ION-treated macrophages at the test concentration (Figure S9). We have tested common cytokines secreted by macrophages such as IL-6, IL-12, and IL-1β and did not find significant stimulatory effects with ION (Figure S10). Further research is needed to elucidate the mechanisms underlying ATP and HMGB1 secretion. Other cytokines present in macrophage secretions may influence immune responses and should be studied. In this proof-of-concept study, we investigated bare nanoparticles to minimize the influence of surface coatings on macrophage stimulation and function, which could complicate comparisons among metal oxide nanoparticles. These nanoparticles lack the long circulation half-life needed for sufficient tumor accumulation. In future studies, it would be valuable to explore surface-modified iron oxide nanoparticles targeted to macrophages that can be administered intravenously.

We studied radiotherapy as a representative treatment to assess the impact of ION-loaded macrophages on the TME. While radiation can induce ICD, its efficacy is dose-dependent and radiation therapy alone is often insufficient to induce a potent immune response.^50^ Agents capable of enhancing ICD of cancer cells or directly targeting and activating DCs have been explored to augment radiation-induced immunity.^9,51,52^ The current study suggests the potential of using inorganic nanoparticles to target tumor macrophages and modify the TME, which represents a different approach. Future research should explore the optimal radiation dose for combination with nanoparticles to achieve the best therapeutic outcomes. It is worth noting that the treatment benefits were seen when macrophages were used in conjunction with radiation, possibly because radiation is required to induce tumor antigen secretion and surface exposure of CRT. Therefore, ION or ION macrophages are more effective as an adjuvant rather than as a standalone treatment. Similar to radiation therapy, many chemotherapeutic agents are poor ICD inducers. It is anticipated that the benefits observed here with ION or ION macrophages may also extend to enhance the efficacy of chemotherapy.

## Experimental Section

### Characterization of Nanoparticles

The morphology, size distributions, and zeta potential of three commercially available nanoparticles: Fe_2_O_3_, iron oxide nanopowder (ION, 544884-Aldrich), Al_2_O_3_, aluminum oxide nanopowder (AON, 544833-Aldrich), and ZnO, zinc oxide nanoparticles (ZON, 721077-Aldrich), were characterized by scanning electron microscopy (SEM, FEI Teneo), dynamic light scattering (DLS, Malvern Zetasizer Nano S90), and a Zetasizer (Malvern Nano ZS).

### Cell Culture

All cell lines used in this study were from ATCC. Macrophages RAW264.7, murine breast carcinoma 4T1 cells, and murine colorectal carcinoma cells CT26 were cultured in RPMI-1640 (Corning, 10-040-CV); melanoma cancer cells B16F10-OVA expressing ovalbumin were grown in high-glucose DMEM (ATCC, 30-2002) supplemented with G418 (for positive selection). All culture media were supplemented with 10% fetal bovine serum and 1% penicillin/streptomycin. All cells were maintained at 37 °C with a humidified 5% CO_2_ atmosphere.

### Cytotoxicity of Nanoparticles on Macrophages

RAW264.7 cells were seeded into 96-well plates overnight. After being treated with series concentrations of different nanoparticles and coincubation for 24 h, the cell viability was measured by a 3-(4,5-dimethylthiazol-2-yl)-2,5-diphenyl tetrazolium bromide (MTT) assay to determine the appropriate concentration range for the following test. Absorbance at 570 nm was read by a microplate reader (Synergy Mx, BioTek).

### Loading Macrophages with Nanoparticles

RAW264.7 cells or BMDMs were seeded in 100 mm Petri dishes overnight and then incubated with nanoparticles at the designated concentrations. BMDMs were prepared and cultured as described in a previous study.^53^ For nanoparticle-loaded macrophages intended for injection, the cells were harvested after trypsinization, washed with PBS, and resuspended in PBS. Otherwise, the medium was replaced with a fresh medium, and the cells were further cultured for the subsequent experiments detailed below.

### ATP and HMGB1 Release

After 24 h of incubation post 2 h nanoparticle treatment, the cell supernatant was collected and measured by an ATP 1step luminescence assay to quantify extracellular ATP. The luminescence was measured by a microplate reader, and the ATP amount was quantified using a calibration curve. HMGB1 secretion was quantified by a mouse HMG1/HMGB1 Quant ELISA kit (LS-F23080, LSBio) following the manufacturer’s instructions.

### CRT Expression on Macrophages

After 24 h of incubation with nanoparticles, the macrophages were collected with cell lifters and stained with an Alexa Fluor 647-conjugated anti-CRT antibody (ab196159, Abcam). Surface CRT expression was then assessed by flow cytometry. The data were expressed and compared in terms of mean fluorescence intensity (MFI).

### Macrophage Phenotype Characterization

M1-like markers (TNF-α, CD86, and iNOS) and M2-like markers (CD206 and IL-10) were assessed to characterize the phenotype change of macrophages. Specifically, after 2 h nanoparticle treatment and a further 24 h incubation, macrophages were collected by cell lifters, stained with CD86 (BV605, BioLegend), iNOS (APC, eBioscience), and CD206 (PE, BD Biosciences) antibodies, and then evaluated by flow cytometry. The signals of each group were presented in MFI compared to the control group. On the other hand, cell supernatants were collected at 24 h to quantify the concentrations of different cytokines, including TNF-α and IL-10 by the enzyme-linked immunosorbent assay (ELISA).

### Cytotoxicity of Nanoparticle-Loaded Macrophages on Cancer Cells

The cytotoxicity effect of nanoparticle-loaded macrophages on cancer cells was investigated through transwell plates. 4T1 breast cancer cells were seeded on the lower chamber of the plates overnight. The following day, macrophages were treated with nanoparticles for 2 h at first, and then, macrophages or nanoparticle-loaded macrophages were added to the upper chamber. The transwell membrane (Corning, 0.4 μm pore size) isolates cancer cells from direct contact with macrophages but enables soluble signal molecules secreted by macrophages to pass through. After 24 h of coincubation, cancer cell viability was assessed by the MTT assay, and the apoptosis or necrosis process of cancer cells was determined by the Annexin V-FITC/PI assay through flow cytometry. H_2_O_2_ treatment of cancer cells could serve as a positive control.

### Migration and Activation of BMDCs

BMDCs were derived from bone marrow progenitor cells and prepared according to the previous papers.^54,55^ Transwell (Corning, 8.0 μm pore size) was adopted to study the migration and activation of BMDCs. Briefly, CFSE prestained BMDCs were added to the upper chamber of the transwell, while B16F10-OVA cancer cells (irradiated or unirradiated) were seeded to the lower chamber and incubated with the supernatant collected from nanoparticle-treated macrophages. The cell number ratio between BMDCs and B16F10-OVA cells was set as 1:1. After 24 h, cells in the lower chamber were collected. The CFSE^+^BMDCs population was analyzed by flow cytometry to study the migration ability of BMDCs. As for the activation study, unstained BMDCs were used at the beginning, and after 24 h incubation, cells in the lower chamber were collected and stained with CD11c (PE-Cy7, BD Biosciences), MHC-II (AF488 I-A/I-E, BioLegend), CD86 (BV605, BD Biosciences), CD80 (PerCP-Cy5.5, BD Biosciences), and SIINEFKL (APC OVA257-264, BioLegend). The positive population of each marker of DCs was analyzed by flow cytometry (NovoCyte Quanteon Flow Cytometer Systems 4 Lasers).

### *In Vivo* Immunotherapy Study

All experimental procedures were conducted following protocols approved by the Institutional Animal Care and Use Committee (IACUC) of the University of Georgia and were performed in accordance with ARRIVE guidelines. Twenty female C57BL/6 mice were subcutaneously inoculated with 0.5 M B16F10-OVA cells. After the tumor size reached 50 mm^3^, the mice were randomly divided into four groups and given treatment as follows: (1) PBS, (2) IR (5 Gy), (3) IR+MF, and (4) IR+MF-ION. After 7 days, tumors were collected, minced into small pieces, and digested to make single-cell suspensions with homemade digestion solution (1 mg/mL collagenase IV, 150 U/mL DNase I, GlutaMax (1x), and 1% P/S in an RPMI-1640 medium) and cell strainers (100 μm pore size). Multiple antibodies were used to stain the cells to evaluate the activities of immune cells in the tumor microenvironment. In a separate experiment, 2 M splenocytes obtained from spleens of euthanized mice were coincubated with 0.5 M B16F10-OVA cells in the presence of a Golgi inhibitor. After 4 h, cells were collected, stained with CD8 and CD4, followed by fixation and permeabilization, and further stained with IFN-γ. The CD8^+^ and CD4^+^ population of T cells and the percentage of IFN-γ among CD8^+^ T cells were quantified by flow cytometry.
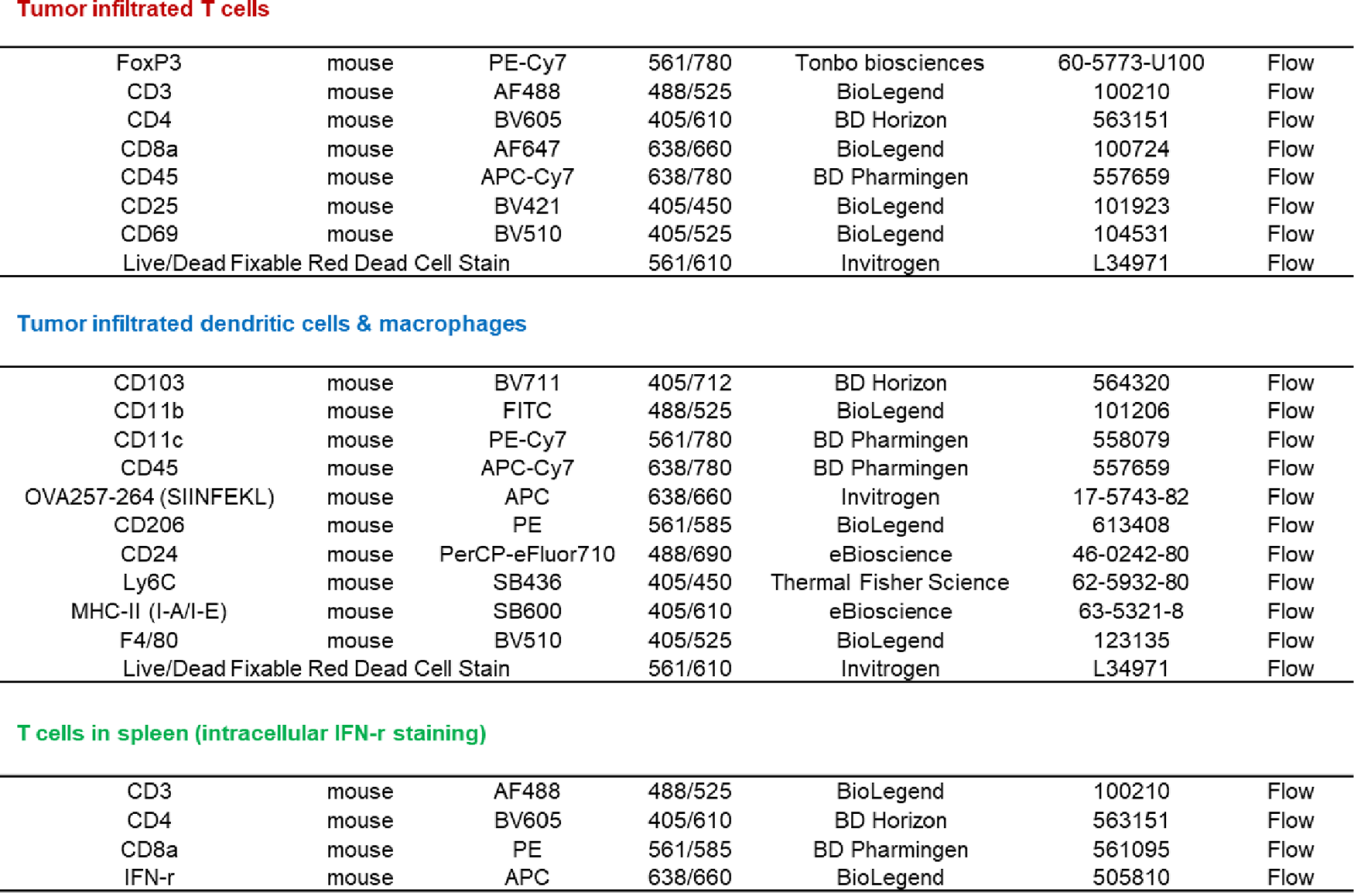


### Therapy Studies

CT26 colorectal carcinoma cells were subcutaneously inoculated on the right hind leg of Balb/c mice. Treatments started when the tumor size reached ∼100 mm^3^. Fifteen mice were randomly divided into five groups and were treated with radiotherapy (IR) followed by i.t. injection of PBS, MF, MF-ION, MF-AON, and MF-ZON. Nanoparticle-loaded macrophages were prepared and collected post 2 h of incubation with nanoparticles, ION-40 μg/mL, AON-80 μg/mL, and ZON-5 μg/mL. Two separate treatments, ∼250,000 cells in 100 μL of PBS/mouse and ∼500,000 cells in 100 μL of PBS/mouse, were given 7 days apart. The tumor size and body weight were monitored every other day. The tumor volume was calculated by the following equation: tumor volume = length × (width)^2^/2, where length ≥ width. Mice were euthanized once the tumor volume was above 1700 mm^3^ by exposure to CO_2_.

In separate studies, murine B16F10-OVA melanoma cancer cells (H2b) were subcutaneously inoculated on the right flank of C57BL/5 mice. Treatments started when the tumor size reaches ∼50 mm^3^. Three treatments were given every other 2 days within 1 week. The tumor size and body weight were monitored every other day. Mice were euthanized by exposure to CO_2_ once the tumor volume was above 1700 mm^3^. All tumors and organs including the heart, liver, spleen, lung, and kidney were harvested for histopathology analysis, basically H&E and *K*i-67 staining.

### Statistical Methods

Quantitative data were expressed as means ± SEM or means ± SD, as detailed in the figure captions. One-tailed Student’s *t* test and one-way ANOVA were used for statistical comparison between experimental groups and control groups for different studies. GraphPad Prism 9 software (San Diego, CA) was used for data analysis. *p* < 0.05 was considered statistically significant.

## Data Availability

The data that support the findings of this study are available from the corresponding author upon reasonable request.

## Supporting Information Available

Supplementary figures on nanoparticle characterization, flow cytometry gating strategies, M1/M2 macrophage ratio post treatment, and H&E staining results (PDF)
