# Supplementary material for: Iron Oxide Nanoparticles Induce Macrophage Secretion of ATP and HMGB1 to Enhance Irradiation-Led Immunogenic Cell Death
Source: Bioconjug Chem. 2024 Dec 16;36(1):80–91. doi: 10.1021/acs.bioconjchem.4c00488 (PMC11740999; doi:10.1021/acs.bioconjchem.4c00488)
Supplement: Supplementary file 1 — bc4c00488_si_001.pdf [file bc4c00488_si_001.pdf]

Supporting Information for

Iron Oxide Nanoparticles Induce Macrophage Secretion of ATP and HMGB1 to Enhance Irradiation-Led Immunogenic Cell Death

Shuyue Zhan[†,§], *Zhengwei Cao*[†,§], *Jianwen Li*[†], *Fanghui Chen*[‡], *Xinning Lai*[†], *Wei Yang*[†], *Yong Teng*[‡], *Zibo Li*[//], *Weizhong Zhang*[†,*], *Jin Xie*[†,*]

[†] Department of Chemistry, University of Georgia, Athens, GA 30602, USA.

[‡] Department of Hematology and Medical Oncology & Winship Cancer Institute, Emory University School of Medicine, Atlanta, GA, 30322, USA

[//] Department of Radiology, Biomedical Research Imaging Center, and Lineberger Comprehensive Cancer Center, University of North Carolina at Chapel Hill, Chapel Hill, North Carolina 27599, USA

* Corresponding authors:

Weizhong Zhang, bryan.zhang.weizhong@gmail.com

Jin Xie, jinxie@uga.edu

Supplementary Figures

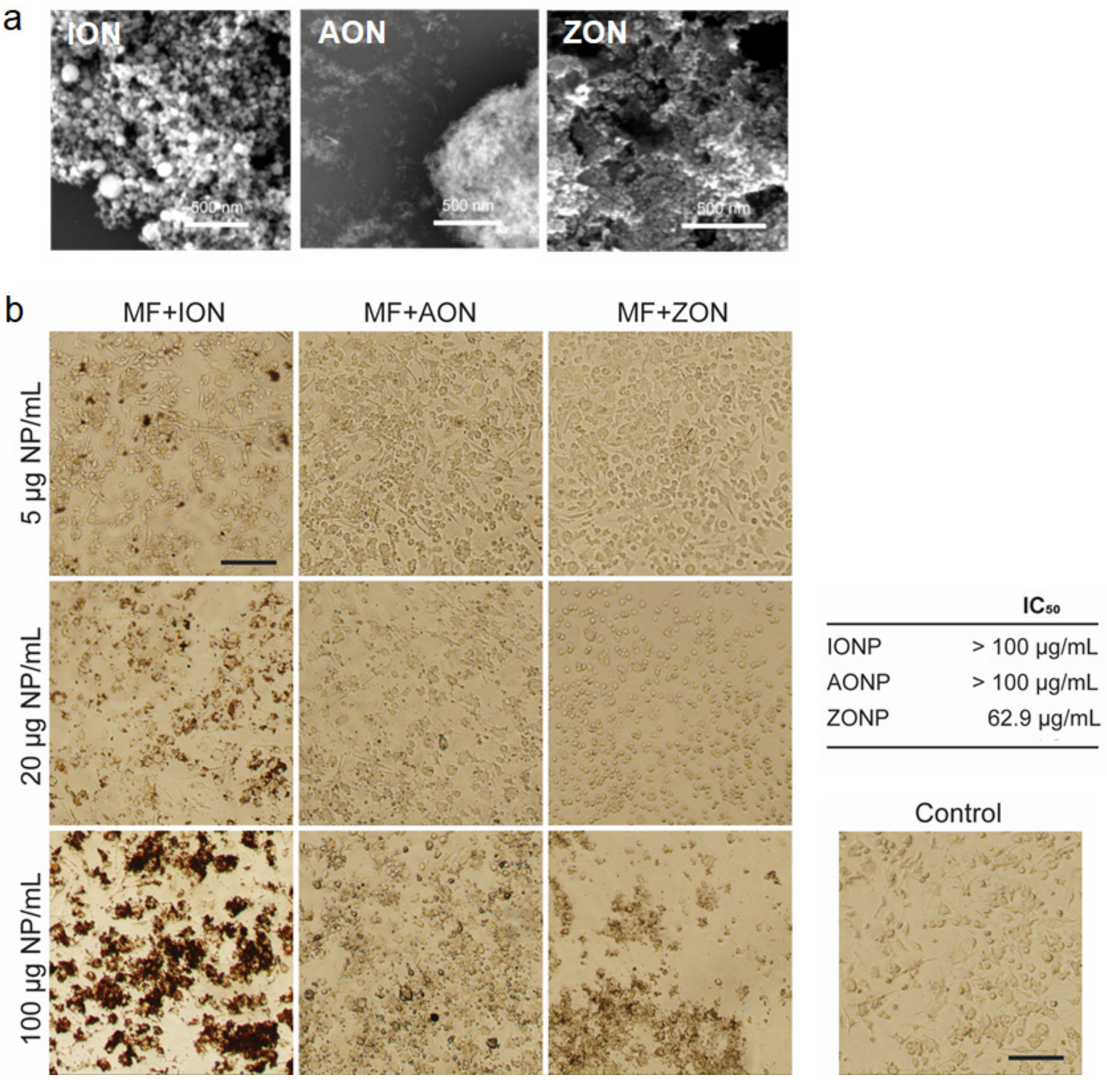

Figure S1. Nanoparticle characterization. a). SEM images of ION, AON, ZON. b). The morphology of macrophages (RAW264.7) when treated with serials concentration of nanoparticles. Scale bar: 50 µm.

	Z-Average	Intensity	Volume
ION	103.8	115.1	83.42
AON	199.7	144.3	137.0
ZON	235.6	124.6	124.6

Figure S2. Additional DLS analysis of the nanoparticle solutions, which are based on intensity and volume distribution.

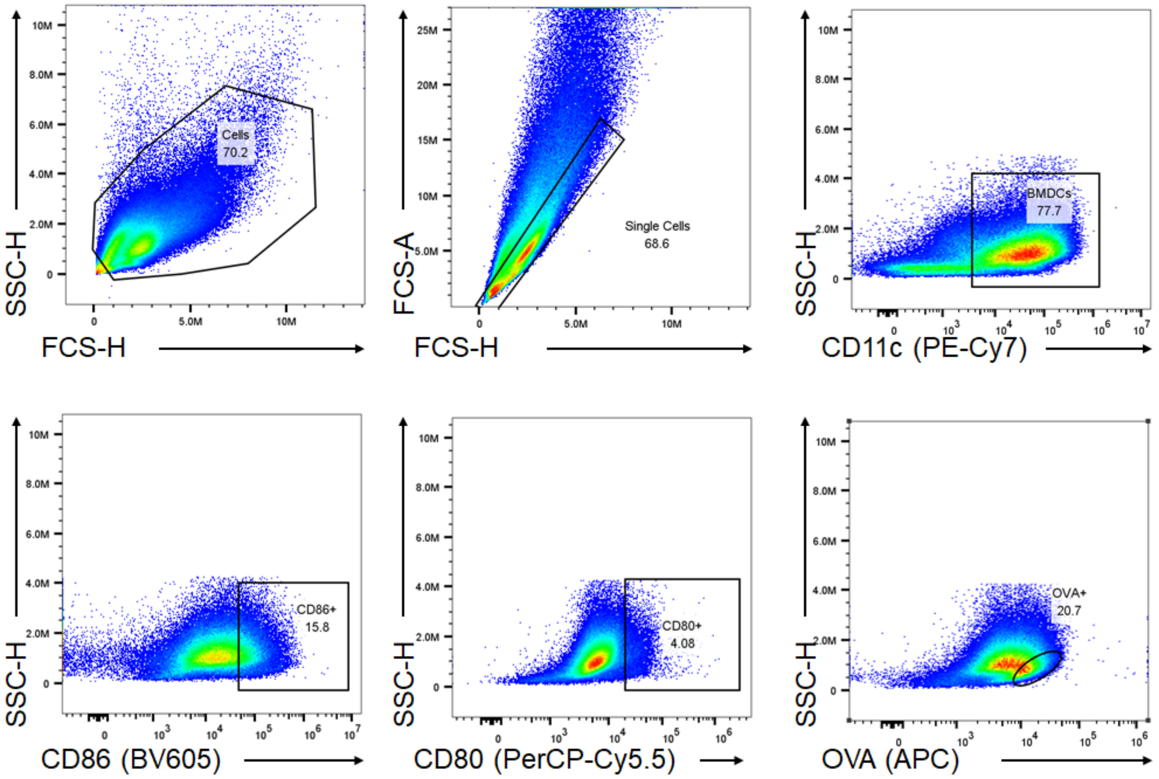

Figure S3: Gating strategy for studying maturation of bone marrow derived dendritic cells (BMDCs). The gating threshold was determined by fluorescence minus one (FMO) control.

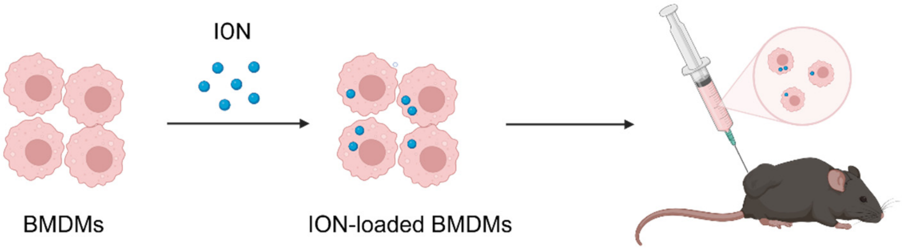

Figure S4. Schematic illustration of the preparation of ION-loaded macrophages.

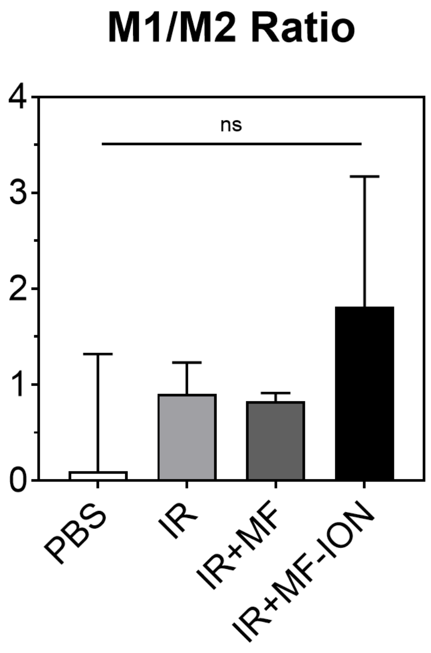

Figure S5. M1 type macrophage to M2 type macrophage ratio.

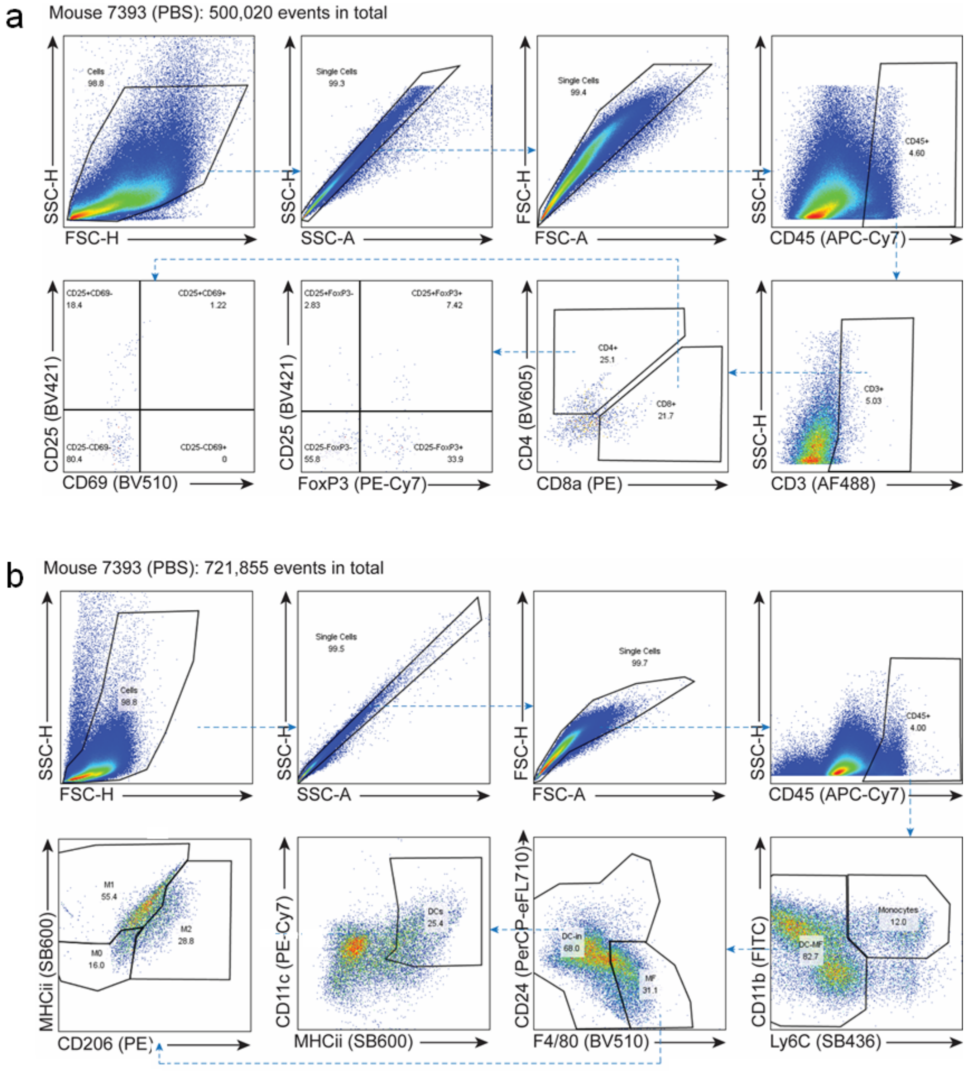

Figure S6. Gating strategy demonstration. a). Tumor-infiltrating T cells. b). Tumor-infiltrating macrophages and dendritic cells.

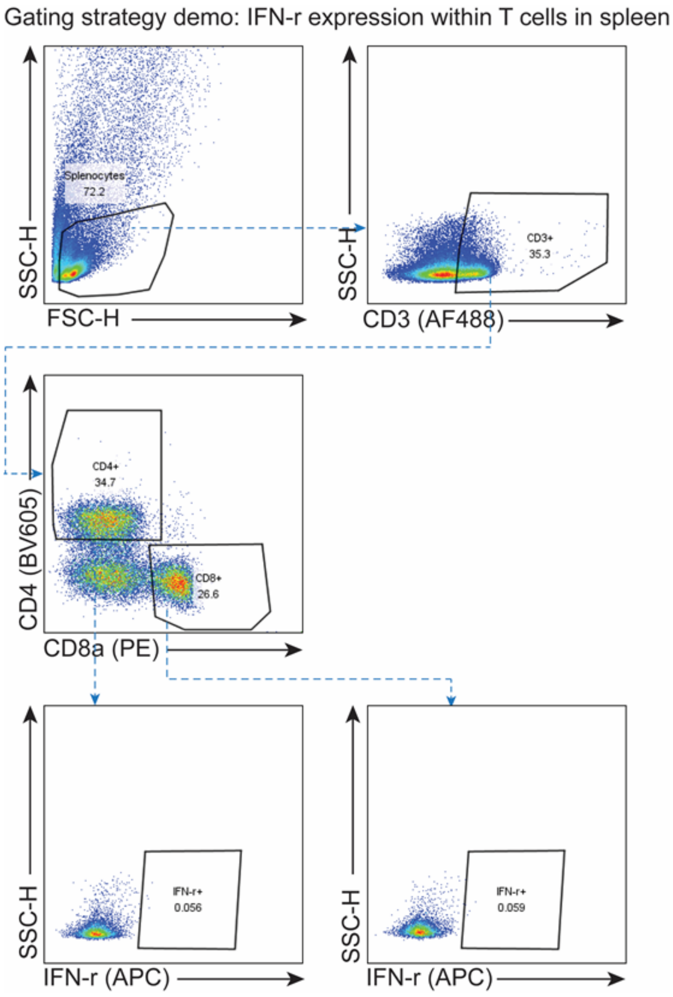

Figure S7. Gating strategy. IFN-γ expression within T cells in splenocytes.

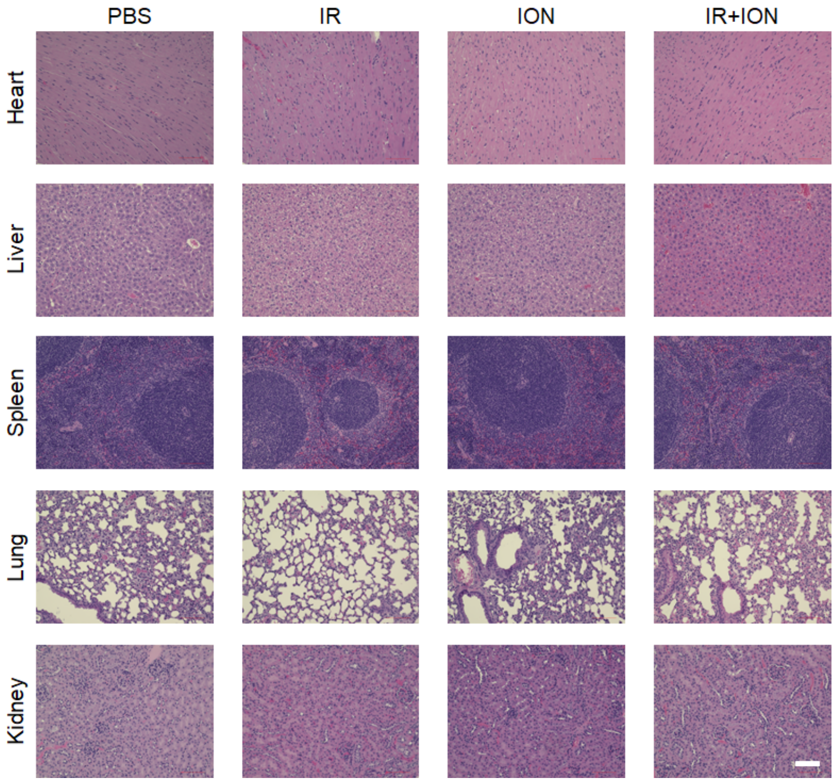

Figure S8. H&E staining of major organ tissues. Sample were taken from B16 tumor-bearing C57BL/6 mice treated with a combination of ION and irradiation (IR). PBS, IR alone, and ION alone were also tested. Scale bar: 100 µm.

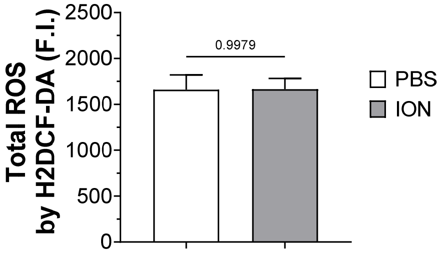

Figure S9. Cellular ROS levels, measured by H2DCF-DA in RAW264.7 cells treated with PBS or ION.

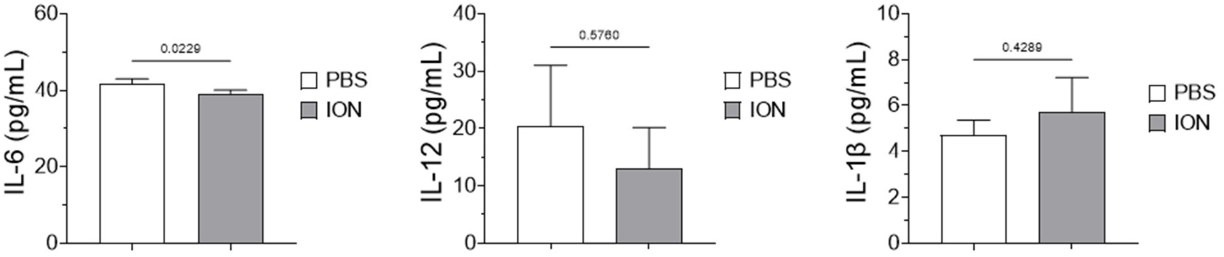

Figure S10. Secretion of IL-6, IL-12, and IL-1β from macrophages treated with PBS or ION, measured by ELISA.
